# Bacterial Poisons

**Published:** 1891-10

**Authors:** 


### Bacterial Poisons.

The author sums up the present state of our knowledge on the above subject as follows:

1. Man is attacked by the infectious diseases either through the alimentary canal or through the blood or lymph.

2. The gastric juice is a physiological guard against infection by the way of the intestines.

3. Additional guards against infection by the intestines are probably to be found in the absorbing cells of the stomach and intestines.

4. Susceptibility to the intestinal infectious diseases is increased when, for any reason, these physiological guards are defective.

5. All toxicogenic germs are dangerous when introduced into the intestines, and their capability of doing injury lies in their production of chemical poisons.

6. Many of these poisons are proteid in character.

7. These poisonous proteids most probably act by catalysis.

8. In the splitting up of complex molecules into simpler ones, heat is liberated and fever manifests itself.

9. The physiological guard against infection through the blood or lymph lies in the germicidal action of the proteids of these fluids.

10. Susceptibility to infection through the blood or lymph is increased by impoverishment of these fluids.

11. We can continue to treat consumption and other systemic diseases by the employment of liberal diet, exercise in the open air, and constitutional remedies without being unscientific in our practice.

12. Filth, without being the bearer of a specific germ, is a cause of disease.

13. Wherever man pollutes the soil about him, the air which he breathes, and the water he drinks with his own excretions, there enteric fever will be found.

14. In their causal relation to disease germs can not be classified without a knowledge of the chemical changes which they induce.

15. While certain bacterial poisons can result only from the growth of certain germs, other poisons similar to one another in their action, though probably not identical, may result from any one of a number of organisms. In the former case we have such diseases as anthrax and small-pox, with their practically constant symptoms and well-marked course; in the latter case we have such diseases as the summer diarrhœa of infancy and enteric fever, with their varying symptoms.—VICTOR C. VAUGHAN, *Med. News.*

